# Differential associations between simple physical performance tests with global and specific cognitive functions in cognitively normal and mild cognitive impairment: a cross-sectional cohort study of Asian community-dwelling older adults

**DOI:** 10.1186/s12877-022-03434-4

**Published:** 2022-10-13

**Authors:** Ted Kheng Siang Ng, Madeline Fu Yun Han, Ping Yeap Loh, Ee Heok Kua, Junhong Yu, John R. Best, Rathi Mahendran

**Affiliations:** 1 Department of Psychological Medicine, Yong Loo Lin School of Medicine, National University of Singapore, NUHS Tower Block, 1E Kent Ridge Road, Singapore, 119228 Singapore; 2 Edson College of Nursing and Health Innovation, Arizona State University, Health North, Suite 301, 550 N 3rd Street, Phoenix, AZ 85004 USA; 3 Department of Human Science, Faculty of Design, Kyushu University, Shiobaru 4-9-1, Fukuoka, 815-8540 Japan; 4 Department of Psychological Medicine, National University Hospital, NUHS Tower Block, 1E Kent Ridge Road, Singapore, 119228 Singapore; 5 Psychology, School of Social Sciences, Nanyang Technological University, 50 Nanyang Avenue, Singapore, 639798 Singapore; 6 Gerontology Research Centre, Simon Fraser University, 515 W Hastings St, Vancouver, B.C V6B 5K3 Canada

**Keywords:** Neurocognitive tests, Executive function, Memory, Mild cognitive impairment, Preclinical dementia, Moderating effect, Timed-up-and-go test, Gait speed, Asian

## Abstract

**Background:**

Physical performance declines and executive dysfunctions are predictors of dementia. However, their associations are not well understood in Asian older adults without dementia (cognitively normal [CN] and mild cognitive impairment [MCI]), especially in a single study.

**Objective:**

Examine the associations between physical performance measures with executive function (EF)-based and non-EF-based neurocognitive tests and whether preclinical dementia cognitive status i.e., CN and MCI, moderated these associations.

**Methods:**

We examined cross-sectional cohort of 716 community-dwelling older adults without dementia (CN = 562 and MCI = 154) using multivariable linear regression models. We associated three simple physical performance measures, namely timed-up-and-go (TUG), fast gait speed (FGS), and 30-s chair stand test (30 s-CST), with a comprehensive neurocognitive test battery measuring EF and non-EF cognitive functions. Moderating effects of cognitive status on the associations were examined. In all models, we controlled for pertinent covariates, including age, education, medical and psychiatric status.

**Results:**

Upon controlling for covariates, TUG was most strongly and positively associated with multiple EF-based neurocognitive tests, followed by FGS, with 30 s-CST having the weakest associations. For all physical performance measures, no significant associations with non-EF-based neurocognitive tests were detected. Cognitive status significantly moderated the associations between all physical measures and several neurocognitive tests, with stronger associations in the MCI than CN.

**Conclusion:**

Compared to FGS and 30 s-CST, TUG had the most robust associations with multiple EF-based cognitive functions. Given their differential associations with global and detailed neurocognitive tests and significant moderating effects of cognitive status, findings highlight a need to carefully consider the choices of simple physical performance tests when using these tests with a heterogenous group of community-dwelling older adults without dementia.

**Supplementary Information:**

The online version contains supplementary material available at 10.1186/s12877-022-03434-4.

## Introduction

Mild cognitive impairment (MCI) represents a preclinical transition from healthy cognitive functioning to dementia [1]. Compared to healthy older adults without cognitive impairment (CI), impaired physical performances are common and are significant predictors of dementia in a disease-stage-dependent manner [2–4]. Physical performance impairments are often co-morbid with cognitive impairment at the preclinical dementia stage (i.e. MCI). It is unsurprising, as motor control and cognition are controlled by prefrontal cortices, fronto-parietal, and cingulate regions of the brain, which are vulnerable to age-related pathologies, and vascular and neurodegenerative diseases [5–7]. Given the shared neural mechanisms between physical performance and cognition, understanding the associations between physical performance tests and cognition can lead to improved dementia risk prediction and early interventions.

There are various physical performance tests with high validity to test physical performance of older adults, often with varying difficulty levels, thus placing different levels of physical and cognitive demands on the test participants. Several complex physical performance measures—e.g., stride length, stride time variability, and dual motor-cognitive tasks—require specialized laboratory equipment, which limits their application in community settings where most older adults without dementia reside in. In contrast, simple physical performance tests, including timed-up-and-go (TUG), fast gait speed (FGS), and 30-s chair stand test (30 s-CST), are valid and easy to administer within a wide range of clinical and community settings—especially in large-scaled community screening programs—and among adults with and without cognitive impairment [8–11].

When considering potential associations between physical performance and cognition, it is important to recognize that cognition involves several distinct abilities or processes. A critical aspect of cognition is executive function (EF), which involves a set of interrelated higher-order cognitive abilities that regulate thoughts, actions, and behaviours [12, 13]. Although related, EF can be considered conceptually and neurobiologically distinct from other aspects of cognition, including learning and memory [14]. However, distinct cognition functions often work in concert when completing a task. For example, working memory is required to retain newly learned information while executing other tasks, including learning, reasoning, and comprehension [15]. Similarly, the execution of a single physical performance test could engage multiple cognitive domains, and it is possible that impairment in physical performance could be evident prior to cognitive decline. A longitudinal study indicated that slow gait speed predicted decline in cognition, especially EF, to a greater degree than poor cognition predicting gait slowing [16]. Another study suggested that gait slowing precedes the diagnosis of MCI [17]. This directional effect may be explained by the occurrence of neurodegeneration, including white matter degeneration, in brain regions that subserve cognition and motor control [18]. Physical performance may be more sensitive to such neurodegeneration prior to cognitive performance. Thus, examinations of the associations between cognitive and physical performance tests may identify preclinical markers and improve screening and diagnostic assessments [10].

Extant literature has shown significant associations between *complex* physical performance and neurocognitive tests, specifically EF-based cognitive tests. For example, stride time variability is associated significantly with multiple cognitive measures [19]. However, several gaps in knowledge in the literature persist. First, studies examining the associations between *simple* physical performance measures, including the TUG, FGS and 30 s-CST, and cognitive functions in older adults, especially of Asian descendants, are relatively scarce. Second, there is a lack of studies contrasting these associations in cognitively normal (CN) versus individuals in the preclinical dementia stage (MCI), with the few studies that have examined such associations *in separate studies* presenting inconsistent findings [20–23]. Specifically, despite diagnostic group-based differences in physical performance measures demonstrated in different stages of cognitive impairment in older adults [24] and stage-dependent declines in cognitive abilities, very few studies have examined the associations between simple physical performance measures and executive functions (EFs), directly comparing older adult with and without MCI within *a single study*. Older adults with altered physical performance measures, i.e. those diagnosed with MCI, have increased reliance on EFs as physical performance tasks become increasingly difficult to execute as one’s condition progresses [5, 20]. As a result, EF and physical performance could be more tightly coupled among individuals with cognitive impairment. Amongst the very few studies examining physical performance measures and cognition in older adults with MCI/ Alzheimer’s disease (AD) and compared them with CN, they were limited by small sample sizes, were conducted in the clinical settings, and in Western countries/populations, thus limiting the generalizability of the findings [6, 25, 26]. Third, most extant studies focused on examining the associations between physical performance measures with either the global cognition score [27], such as the Mini-Mental State Examination (MMSE), *or* a limited battery of neurocognitive tests *exclusively* assessing executive functions [16, 28, 29]. This issue raised the question on whether cognitive processes *other than* executive functions are also associated with simple physical performance measures. Furthermore, whether there is a single physical performance test that has significant associations with *both* global cognition and specific cognitive domains is also unclear. Hence, previous studies with frequently isolated examinations of the relationships between complicated physical performance measures within a single diagnostic entity at the late disease stage (i.e. AD) hinders a more comprehensive and holistic understanding of the nuanced relationships between physical performance and different cognitive processes in the preclinical AD stages [5]. Further investigation on whether cognitive status modifies the associations between multiple simple physical performance and a comprehensive battery of neurocognitive tests is thus warranted.

Taken together, there is a gap in knowledge on investigations of simple physical performance measures and their differential associations with global cognition and comprehensive neurocognitive tests, encompassing the EF and non-EF cognitive domains, within a single study in Asian older adults, and whether these associations are moderated by cognitive status. To this end, analyzing the Community Health and Intergenerational (CHI) cohort, a community-based cohort study based in Singapore [30], we aimed to investigate:

1. Differential associations between simple physical performance tests and a) global cognition, b) EF-based, and c) non-EF-based cognitive domains;
2. Hypothesized differences in the associations with global, EF-based, and non-EF-based cognitive domains, based on the different simple physical performance tests;
3. Whether preclinical dementia cognitive status (i.e., CN/MCI) moderated the associations between simple physical performance and neurocognitive tests.

## Methods

### Participants and procedures

The participants were part of a larger cohort study: CHI. The CHI study is an ongoing study involving community-dwelling older adults in Singapore, aiming to investigate the biological, psychological, and social aspects of the aging process [30]. The inclusion criteria for the CHI study were older adults aged between 60 and 99 years. Participants were recruited via door-to-door visits in the Western region of Singapore or by word of mouth.

Data for 831 participants were available. Specifically, for our research aims in this paper, we established the following eligibility criteria for data analysis. First, participants must have completed their neurocognitive assessments and were not diagnosed with dementia. Neurocognitive diagnoses were performed via conducting a consensus meeting of psychiatrists and psychologists who reviewed participants’ scores comprising the Clinical Dementia Rating Scale, Modified Mini-Mental State Examination (MMSE), and the neurocognitive battery, which would be described subsequently. Cognitive statuses of CN, MCI or dementia were determined based on Peterson’s criteria for MCI [31] and the Diagnostic and Statistical Manual of Mental Disorders, DSM-V criteria for Major Neurocognitive Disorder (dementia) [32]. Those with Parkinson’s disease and other self-reported neurological disorders were also excluded from the analysis. Participants must have completed at least one of the three physical performance measures. These exclusion criteria resulted in a final sample size of 716 participants in this study (CN = 562; MCI = 154).

### Measures

#### Physical performance measures

Assessors ensured the following before the start of each physical performance measure test: participants were physically well, wore their regular footwear, and had their assistive devices at hand if required. Participants were allowed to stop if they experienced fatigue when executing the test(s). If they still wished to continue, participants could arrange to finish the remaining measure(s) on a different date. Participants were not tested beyond their limits, and assessors were present throughout all test measures to ensure safety.

The three physical performance measures, the TUG, FGS and 30 s-CST, takes minimal time and space during testing. TUG is an easy tool to assess mobility and overall physical function in older adults [33, 34], and demonstrates high predictive utility for future institutionalization, falls, and mortality [35–37]. Gait speed, or measuring how long it takes for a participant to walk a short distance, is a widely used measure of functional capacity in healthy and clinical older adult populations [9, 38]. Gait speed has been used as a predictor of decline in functional mobility and is also associated with a range of health-related outcomes, such as frailty, falls, and cognitive decline [39, 40]. While the current literature suggests that protocols for gait speed tests differ greatly [9, 38], we chose to examine fast gait speed (FGS) in our study, measured over a distance of six meters, with the inclusion of both acceleration and deceleration. Lastly, 30 s-CST examines functional lower extremity strength and endurance in older adults [11, 41], and is a predictor of risk for falls [41]. For TUG and FGS, a higher score indicates lower performance. Whereas for 30 s-CST, a higher score indicates higher performance.

There are important differences among the three physical performance measures. 30 s-CST measures specifically lower body ability, whereas TUG and FGS examine functional mobility, with scores associated with various health-related outcomes. Gait and walking [42] ability have been increasingly recognized not only to associate with the musculoskeletal system, but also neurocognitive ability. However, TUG is a more complicated test than FGS—completion of TUG requires more complex motor performance sequences from the test subject than FGS [42, 43]. The detailed procedures and scoring methods for each physical performance measure are described in the supplementary text.

### Neurocognitive tests

#### Global cognition—MMSE

MMSE is a brief 30-point cognitive screening tool, which assesses global cognitive function. The score ranges from zero to 30, with a higher score indicating a higher cognitive performance. This study used English and Mandarin-translated and modified versions, which had been adapted to local cultural contexts, with the norms validated in the Singaporean population [44].

#### Detailed neurocognitive tests measuring specific cognitive domains

We administered a neurocognitive battery of tests, which have previously been validated and utilized in the current population [45]. These tests evaluate specific cognitive functions, including attention, learning, memory, motor speed, and executive functions [46]. We included: 1) Rey Auditory Verbal Learning Test (RAVLT), 2) Digit Span Forward and Backward Task, 3) Color Trails Test (CTT) 1 and 2, 4) Block Design Test, and 5) Semantic Fluency (Animal) Test. RAVLT, Block Design Test, and Semantic Fluency (Animal) Test are non-EF-based neurocognitive tests, while the rest of the tests are EF-based tests. Supplementary Table 1 describes each neurocognitive test, the cognitive domain(s) assessed, and its task descriptions in detail. For all cognitive tests, a higher score indicates better cognitive performance, except for only Color Trails Test (CTT) 1 and 2, in which a higher score indicates lower cognitive performance.

### Covariates

Covariates comprised age, sex, years of formal education, depressive and anxiety symptoms, body-mass index, consumption of prescription medicine, the total number of morbidities, physical activity level, smoking status, and alcohol consumption [47, 48]. We assessed depressive and anxiety symptoms employing the 15-item Geriatric Depression Scale (GDS) and the 20-item Geriatric Anxiety Inventory (GAI). We measured physical activity levels by using the International Physical Activity Questionnaire (IPAQ)’s standard scoring protocol [49]. Details of the measures are available in the supplementary text.

### Statistical analyses

We compared differences in demographic characteristics in the CN and the MCI groups using independent t-tests or chi-squared tests, whenever appropriate. Independent t-tests were performed to test the differences in the physical performance and neurocognitive tests in CN compared to mild cognitive impairment. To investigate the differences in physical performance tests between CN and MCI, further controlling for covariates, we employed linear regression models with cognitive status as the independent variable (MCI, with CN as the reference group) and the physical performance tests as the dependant variables, separately. Supplementary Table 2 details the findings.

To examine aims 1 and 2, investigating the associations between physical performance and neurocognitive tests, we ran linear regression models with the physical performance tests as the independent variable and the neurocognitive tests as the dependant variables. We presented two analytic models, one model unadjusted and a subsequent model fully adjusted for covariates.

To investigate aim 3, examining the moderating effect of cognitive status on the associations between physical performance and neurocognitive tests, all the linear regression models were built on top of the respective fully adjusted models from aim 2, with the addition of an interaction term between physical performance tests and cognitive status.

We presented all the findings addressing aims 1, 2 and 3 in two parts, with the EF-based neurocognitive tests presented in Tables 3, 4, 5 and 6, and the findings for the non-EF-based neurocognitive tests presented in Supplementary tables 3 and 4. All the analyses were performed using Statistical Package for the Social Sciences (SPSS) version 24.0 (IBM SPSS Statistics for Windows, Version 24.0). To correct for multiple testing, especially for measures with high correlations like those examined in our study, we performed the Benjamini–Hochberg Procedure. Considering the likelihood of type I against type II error, we applied a false discovery rate (FDR) of 0.10 correction to the observed p-values across the statistical tests corresponding to those answering our three a priori aims. A final FDR-corrected p-value of 0.038 and below was considered statistically significant.

## Results

### Demographic characteristics

Table 1 summarizes the demographic characteristics of the total study participants (*N* = 716), comparing participants in CN = 562 versus MCI = 154. They have similar mean ages in both groups, with (mean ± SD) CN = 67.94 ± 5.65 and MCI = 68.27 ± 7.03. Most participants were women, with n (%) CN = 378 (67.3%) and MCI = 95 (61.7%) and highly educated, i.e., years of formal education, with (mean ± SD) CN = 13.21 ± 4.20 and MCI = 12.47 ± 4.40. Other demographic characteristics did not differ according to the cognitive status, except depressive symptoms higher in the MCI group (MCI: mean = 1.57, SD = 2.56; CN: mean = 0.95, SD = 1.63, *p* = 0.005). Table 2 shows that of the three physical performance tests, only the TUG significantly differed in CN versus MCI (MCI: mean = 9.99, SD = 2.52; CN: mean = 9.43, SD = 2.35, *p* = 0.010). For the global and detailed neurocognitive tests, all measures except the CTT Interference had significant differences comparing the two groups. Supplementary Table 5. presented the differences in demographic characteristics of participants excluded and included in the sample.

**Table 1:** Demographic characteristics of cognitively normal versus mild cognitive impairment

Demographics Characteristics	Cognitively normal;mean ± SD or n (%)	MCI;mean ± SD or n (%)	*P*-values
Sample Size	562 (78.5)	154 (21.5)	
Age (in years)	67.94 ± 5.65	68.27 ± 7.03	0.591
Sex			
Women	378 (67.3)	95 (61.7)	0.212
Men	184 (32.7)	59 (38.3)	
Years of formal education	13.21 ± 4.20	12.47 ± 4.40	0.065
Depressive symptoms	0.95 ± 1.63	1.57 ± 2.56	0.005**
Anxiety symptoms	1.12 ± 2.44	1.60 ± 3.44	0.105
BMI (kg/m^2^)	23.74 ± 3.76	23.80 ± 3.84	0.853
Total number of prescription medicine	2.12 ± 2.72	2.02 ± 2.43	0.694
Total number of morbidities	2.30 ± 1.60	2.34 ± 1.77	0.773
Physical activity levels (MET)	4354.21 ± 5594.17	4294.93 ± 3957.51	0.903
Smoking status			
Yes	8 (1.4)	5 (3.2)	0.167
No	553 (98.6)	149 (96.8)	
Alcohol consumption			
Yes	115 (20.5)	27 (17.5)	0.428
No	446 (79.5)	127 (82.5)	

Notes: *MCI* mild cognitive impairment, *BMI* body-mass index, *MET* Metabolic equivalent of task

* indicates *p*<0.05, ** indicates *p*<0.01, and *** indicates *p*<0.001

**Table 2:** Means and standard deviations of the physical performance and neurocognitive tests in cognitively normal compared to mild cognitive impairment

Demographics Characteristics	Cognitively normal (n=562);mean (SD)	MCI (n=154);mean (SD)	*P*-values
TUG	9.43 (2.35)	9.99 (2.52)	0.010*
FGS	3.67 (0.97)	3.77 (0.93)	0.230
30s-CST	14.60 (4.40)	13.86 (4.84)	0.075
Global Cognition- MMSE	28.3 (1.703)	27.29 (2.353)	<0.001***
Digit span forward	10.41 (2.36)	9.95 (2.32)	0.032*
Digit span backward	7.05 (2.03)	6.12 (1.93)	<0.001***
CTT 1	49.64 (17.19)	61.65 (30.07)	<0.001***
CTT 2	100.54 (28.09)	123.71 (50.48)	<0.001***
CTT Interference	1.13 (0.56)	1.15 (0.63)	0.783
Block Design test	34.99 (9.68)	30.92 (11.71)	<0.001***
RAVLT T1	5.88 (2.02)	4.76 (2.01)	<0.001***
RAVLT T5	12.32 (2.01)	10.78 (2.27)	<0.001***
RAVLT B	5.28 (1.89)	4.82 (1.98)	0.008**
RAVLT T6	10.71 (2.66)	8.29 (2.77)	<0.001***
RAVLT Sum T1-T5	49.57 (8.48)	41.95 (9.44)	<0.001***
RAVLT Delayed Recall	10.82 (2.66)	8.01 (3.24)	<0.001***
RAVLT Recognition Trial	14.12 (1.23)	12.59 (2.25)	<0.001***
RAVLT Recognition Trial – False Positive	2.04 (3.31)	4.26 (5.22)	<0.001***
Semantic Fluency (Animal) Test	18.29 (4.11)	14.91 (4.65)	<0.001***

Notes: *MCI* mild cognitive impairment, *TUG* timed up and go test, *FGS* fast gait speed, *30s-CST* 30-second chair stand test, *MMSE *mini-mental state examination, *CTT* color trail test, *RAVLT* Rey Auditory Verbal Learning Test

* indicates *p*<0.05, ** indicates *p*<0.01, and *** indicates *p*<0.001

Supplementary Table 2. shows the differences or lack thereof in the three physical performance tests between cognitive status (MCI versus CN as the reference group), upon controlling for covariates. Similar to the results shown in Table 2, FGS and 30 s-CST had no significant differences between groups at the bivariate level. Further controlling for covariates did not change the findings. On the other hand, despite TUG being significantly higher in MCI at the bivariate level (β = 0.558, 95% CI = 0.133 to 0.982, *p* = 0.010, R^2^ = 0.9%), upon further controlling for covariates, the association was attenuated (β = 0.345, 95% CI = -0.027 to 0.717, *p* = 0.069, R^2^ = 27.7%).

#### Differential associations between physical performance measures and EF-based and non-EF-based neurocognitive tests

As shown in Table 3, amongst the associations with EF-based cognitive domains, all, except one of the bivariate associations (CTT interference; β = -0.039, 95% CI = -0.083 to 0.005, *p* = 0.080, R^2^ = 0.4%), with TUG, FGS, and 30 s-CST were significant. Upon fully controlling for covariates, several associations remained significant and explained a relatively large portion of variance, particularly associations with the higher-level executive functions. They include associations between higher TUG with lower digit span backward (β = -0.08, 95% CI = -0.148 to -0.011, *p* = 0.023, R^2^ change = 10.7%), higher CTT1 (β = 1.713, 95% CI = 1.057 to 2.369, *p* < 0.001, R^2^ change = 16.6%), higher CTT2 (β = 1.539, 95% CI = 0.445 to 2.633, *p* = 0.006, R^2^ change = 19%), and lower CTT interference (β = -0.032, 95% CI = -0.053 to -0.012, *p* = 0.002, R^2^ change = 3%).

**Table 3:** Associations between physical performance and executive function-based neurocognitive tests

Neurocognitive tests/ physical performance tests	Models	TUG			FGS			30s-CST		
		β (95% CI)	*P-*values	*R*^*2*^change	β (95% CI)	*P-*values	*R*^*2*^change	β (95% CI)	*P-*values	*R*^*2*^change
Global Cognition- MMSE	Unadjusted	-0.214 (-0.27 to -0.157)	<0.001***	0.072	-0.397 (-0.541 to -0.253)	<0.001***	0.040	0.063 (0.032 to 0.094)	<0.001***	0.021
	Fully-adjusted	-0.116 (-0.177 to -0.055)	<0.001***	0.159	-0.144 (-0.295 to 0.006)	0.061	0.179	0.014 (-0.017 to 0.045)	0.376	0.195
Digit span forward	Unadjusted	-0.09 (-0.162 to -0.017)	0.015*	0.008	-0.214 (-0.395 to -0.034)	0.020*	0.008	0.044 (0.005 to 0.083)	0.029*	0.007
	Fully-adjusted	-0.019 (-0.103 to 0.065)	0.654	0.037	-0.06 (-0.266 to 0.146)	0.569	0.037	0.015 (-0.027 to 0.058)	0.481	0.038
Digit span backward	Unadjusted	-0.194 (-0.255 to -0.132)	<0.001***	0.051	-0.39 (-0.544 to -0.235)	<0.001***	0.033	0.063 (0.029 to 0.097)	<0.001***	0.018
	Fully-adjusted	-0.08 (-0.148 to -0.011)	0.023*	0.107	-0.106 (-0.274 to 0.063)	0.218	0.120	0.007 (-0.028 to 0.041)	0.711	0.134
CTT 1	Unadjusted	3.012 (2.399 to 3.625)	<0.001***	0.115	7.371 (5.833 to 8.909)	<0.001***	0.110	-1.108 (-1.452 to -0.764)	<0.001***	0.053
	Fully-adjusted	1.713 (1.057 to 2.369)	<0.001***	0.166	4.586 (2.982 to 6.19)	<0.001***	0.176	-0.492 (-0.828 to -0.156)	0.004**	0.209
CTT 2	Unadjusted	4.514 (3.477 to 5.552)	<0.001***	0.093	11.707 (9.122 to 14.291)	<0.001***	0.100	-1.767 (-2.343 to -1.191)	<0.001***	0.048
	Fully-adjusted	1.539 (0.445 to 2.633)	0.006**	0.190	5.232 (2.56 to 7.904)	<0.001***	0.190	-0.47 (-1.026 to 0.085)	0.097	0.230
CTT Interference	Unadjusted	-0.027 (-0.044 to -0.009)	0.003**	0.012	-0.039 (-0.083 to 0.005)	0.080	0.004	0.013 (0.004 to 0.023)	0.007**	0.010
	Fully-adjusted	-0.032 (-0.053 to -0.012)	0.002**	0.030	-0.051 (-0.102 to -0.001)	0.047	0.031	0.015 (0.004 to 0.025)	0.005**	0.030

Notes: *MMSE* mini-mental state examination, *CTT* color trail test, *TUG* timed up and go test, *FGS* fast gait speed, *30s-CST* 30-second chair stand test, *β* unstandardized beta-coefficient, *95% CI* 95% confidence interval

* indicates *p*<0.05, ** indicates *p*<0.01, and *** indicates *p*<0.001

Unadjusted: bivariate association between physical performance and neurocognitive tests

Fully-adjusted: added age, sex, years of formal education, depressive and anxiety symptoms, body-mass index, consumption of prescription medicine, total number of morbidities, physical activity level, smoking status, alcohol consumption, and cognitive status. For TUG and FGS, a higher score indicates lower performance. Whereas for 30s-CST, a higher score indicates higher performance. For all cognitive tests, a higher score indicates better cognitive performance, except for only Color Trails Test (CTT) 1 & 2, in which a higher score indicates lower cognitive performance

As shown in Supplementary Table 3, the associations with non-EF-based cognitive domains: All except three (i.e., FGS with RAVLT T5 and with RAVLT recognition trial, and 30 s-CST with RAVLT B) bivariate associations were significant. However, upon fully controlling for covariates, none of the associations remained significant.

#### Differential associations between physical performance measures and neurocognitive tests

As shown in Table 3, despite significant associations with MMSE (global cognition) at the bivariate levels across the three physical performance tests, with higher scores on physical performance tests associated with lower MMSE scores (specifically TUG: β = -0.214, 95% CI = -0.27 to -0.157, *p* < 0.001, R^2^ = 7.2%), only TUG remained significantly and inversely associated with it upon controlling for covariates (β = -0.116, 95% CI = -0.177 to -0.055, *p* < 0.001, R^2^ = 15.9%).

As shown in Table 3, for detailed neurocognitive tests, after controlling for covariates, all three physical performance tests were significantly associated with CTT1. On the other hand, there were many other differences in the significant associations between the physical performance with EF-based cognitive tests. After fully controlling for covariates, higher TUG remained associated significantly with several neurocognitive tests, including lower global cognition-MMSE (β = -0.116, 95% CI = -0.177 to -0.055, *p* < 0.001, R^2^ = 15.9%), lower digit span backward (β = -0.080, 95% CI = -0.148 to 0.011, *p* = 0.023, R^2^ change = 10.7%), higher CTT2 (β = 1.539, 95% CI = 0.445 to 2.633, *p* = 0.006, R^2^ change = 19%), and lower CTT interference (β = -0.032, 95% CI = -0.053 to -0.012, *p* = 0.002, R^2^ change = 3%). Whereas for FGS, only CTT2 (β = 5.232, 95% CI = 2.56 to 7.904, *p* < 0.001, R^2^ change = 19%) remained having significant associations after fully controlling for covariates. Lastly, for 30 s-CST, only higher 30 s-CST remained significantly associated with higher CTT interference after fully controlling for covariates (β = 0.015, 95% CI = 0.004 to 0.025, *p* = 0.005, R^2^ change = 3%).

#### Significant moderation of associations by cognitive status

**Fig. 1:**
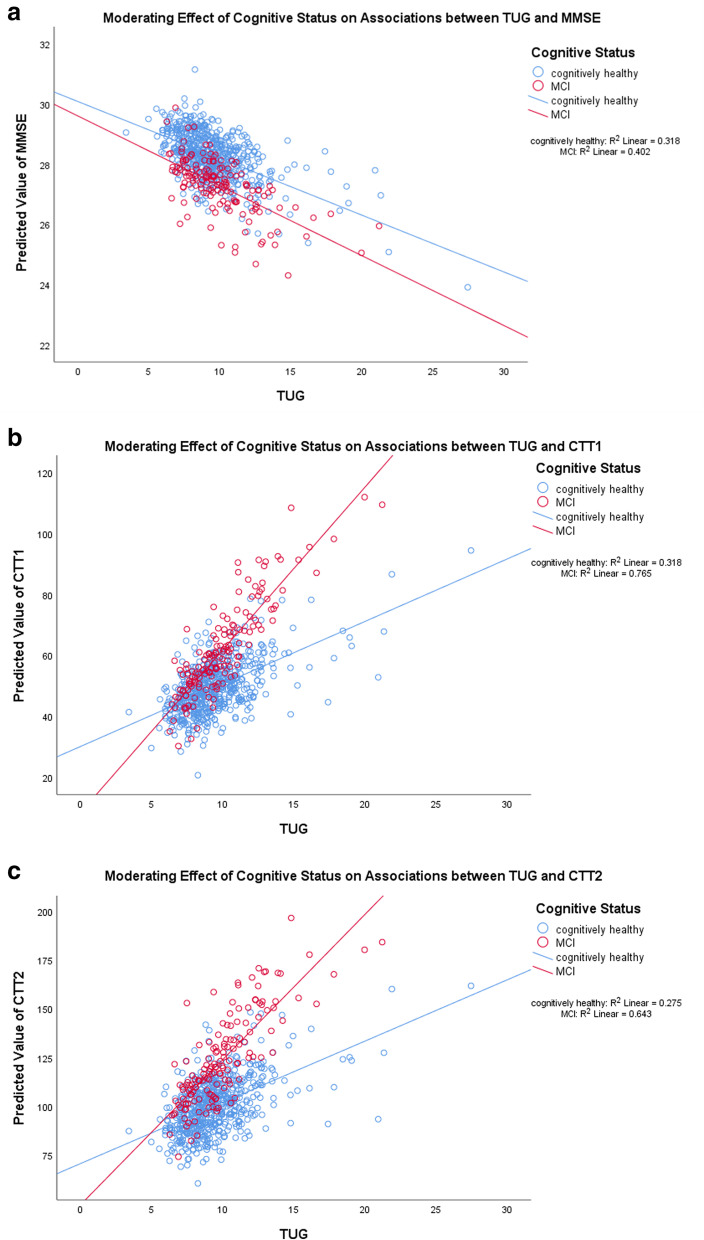
**a**, **b** and **c** Moderating effects of cognitive status on associations between TUG and neurocognitive tests. For TUG, a higher score indicates lower performance. For MMSE, a higher score indicates better cognitive performance, whereas for Color Trails Test (CTT) 1 and 2, a higher score indicates lower cognitive performance

**Fig. 2:**
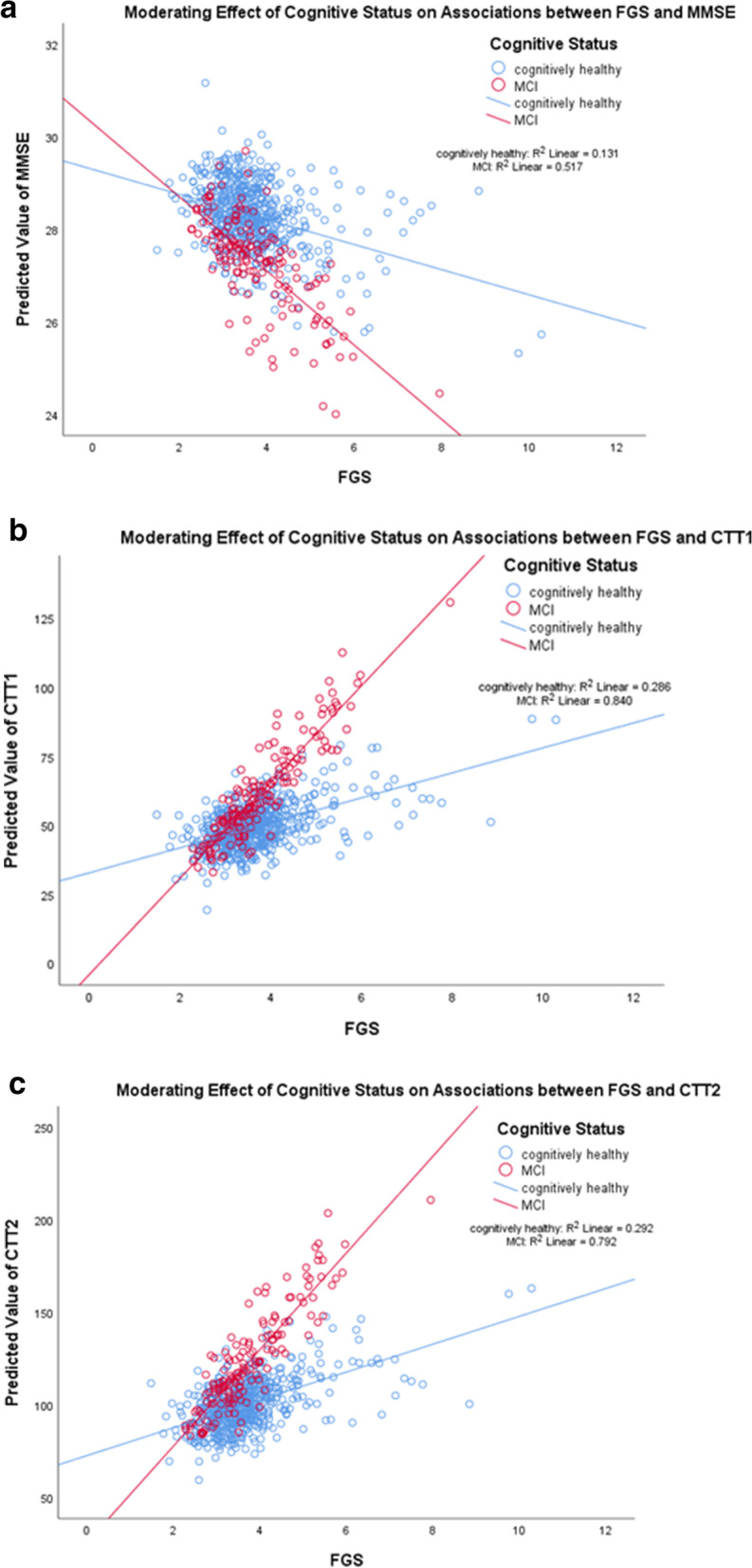
**a**, **b** and **c** Moderating effects of cognitive status on associations between FGS and neurocognitive tests. For FGS, a higher score indicates lower performance. For MMSE, a higher score indicates better cognitive performance, whereas for Color Trails Test (CTT) 1 and 2, a higher score indicates lower cognitive performance

**Fig. 3:**
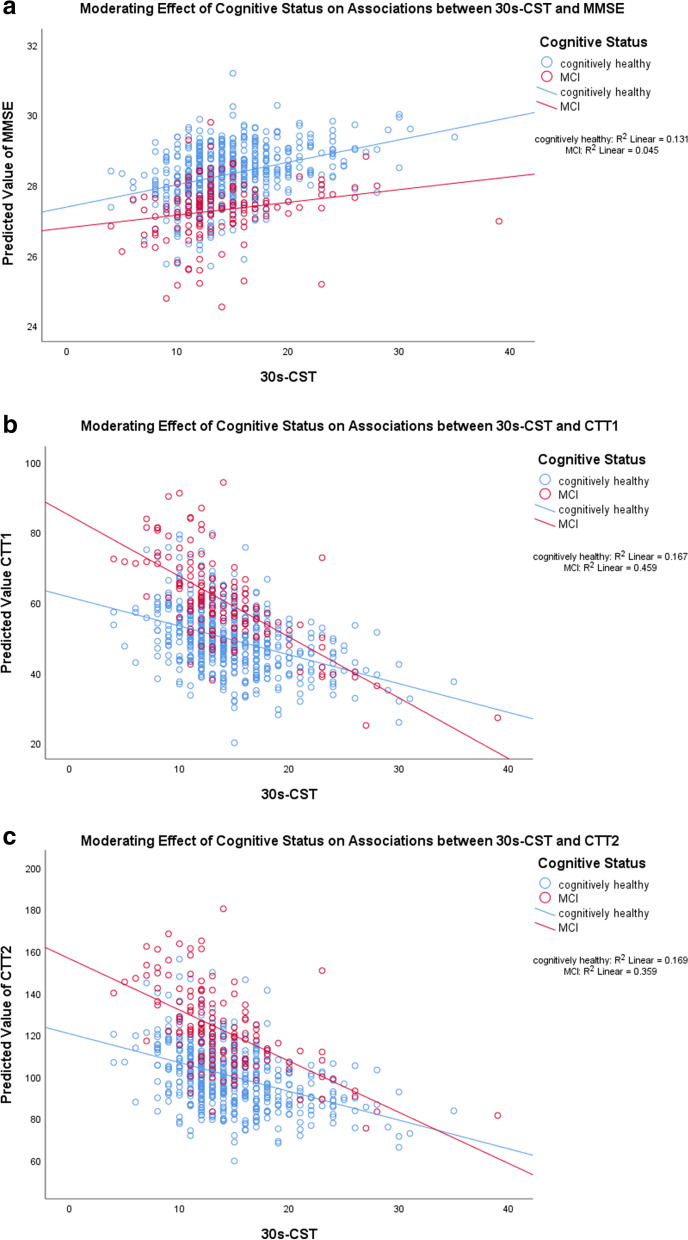
**a**, **b** and **c** Moderating effects of cognitive status on associations between 30 s-CST and neurocognitive tests. For 30 s-CST, a higher score indicates higher performance. For MMSE, a higher score indicates better cognitive performance, whereas for Color Trails Test (CTT) 1 and 2, a higher score indicates lower cognitive performance

For the EF-based cognitive tests, cognitive status moderated the associations between all three physical performance tests with CTT1 (Figs. 1b, 2b and 3b). Furthermore, there were several other significant moderation effect of cognitive status on the associations between the three physical performance measures with other EF-based cognitive tests.

For EF-based cognitive tests, other than CTT1 (β = 2.657, 95% CI = 1.414 to 3.899, *p* < 0.001, R^2^ change = 1.7%), Table 4 and Fig. 1c show that the associations between TUG with one other neurocognitive tests that were stronger in MCI, in that higher TUG was associated with higher CTT2 (β = 3.356, 95% CI = 1.273 to 5.440, *p* = 0.002, R^2^ change = 1%), but not global cognition (Fig. 1a).

**Table 4:** Associations between TUG and executive function-based neurocognitive tests– added interaction term between TUG and cognitive status

Neurocognitive tests/ physical performance tests	TUG Interaction Model						
	TUG		Cognitive Status		TUG x Cognitive Status		
	β (95% CI)	*p*-values	β (95% CI)	*p*-values	β (95% CI)	*p*-values	*R*^*2*^change
Global Cognition- MMSE	-0.089 (-0.244 to 0.066)	0.259	-0.585 (-1.771 to 0.601)	0.333	-0.022 (-0.139 to 0.095)	0.710	0.000
Digit span forward	-0.123 (-0.335 to 0.09)	0.258	-1.154 (-2.786 to 0.477)	0.165	0.085 (-0.076 to 0.246)	0.299	0.001
Digit span backward	-0.114 (-0.288 to 0.059)	0.196	-1.06 (-2.39 to 0.27)	0.118	0.029 (-0.103 to 0.16)	0.67	0.000
CTT 1	-1.512 (-3.154 to 0.129)	0.071	-15.922 (-28.51 to -3.334)	0.013**	2.657 (1.414 to 3.899)	<0.001***	0.017
CTT 2	-2.536 (-5.289 to 0.217)	0.071	-13.195 (-34.309 to 7.919)	0.22	3.356 (1.273 to 5.44)	0.002**	0.010
CTT Interference	-0.009 (-0.061 to 0.043)	0.734	0.214 (-0.183 to 0.612)	0.291	-0.019 (-0.058 to 0.02)	0.336	0.002

Notes: *MMSE* mini-mental state examination, *CTT* color trail test, *TUG* timed up and go test, *FGS* fast gait speed, *30s-CST* 30-second chair stand test, *β* unstandardized beta-coefficient, *95% CI* 95% confidence interval

* indicates *p*<0.05, ** indicates *p*<0.01, and *** indicates *p*<0.001

All the statistical models here were built on top of the respective adjusted models from Table 3, with the addition of an interaction term between physical performance tests and cognitive status. Reference group was the cognitively normal group. For TUG and FGS, a higher score indicates lower performance. Whereas for 30s-CST, a higher score indicates higher performance. For all cognitive tests, a higher score indicates better cognitive performance, except for only Color Trails Test (CTT) 1 & 2, in which a higher score indicates lower cognitive performance

As shown on Figs. 2a, b, and c and Table 5, similarly, associations between FGS and global cognition and two detailed neurocognitive tests were significantly moderated by cognitive status; In MCI, compared to CN, FGS had higher strength of associations with MMSE, CTT1 and CTT 2, in that higher FGS was associated with lower MMSE and higher CTT1 and CTT 2 (β = -0.36, 95% CI = -0.67 to -0.049, *p* = 0.023, R^2^ change = 0.6%; β = 10.78, 95% CI = 7.563 to 13.997, *p* < 0.001, R^2^ change = 4.1%; β = 14.643, 95% CI = 9.228 to 20.058, *p* < 0.001, R^2^ change = 2.7%, respectively).

**Table 5:** Associations between FGS and executive function-based neurocognitive tests– added interaction term between FGS and cognitive status

Neurocognitive tests/ physical performance tests	FGS Interaction Model						
	FGS		Cognitive Status		FGS x Cognitive Status		
	β (95% CI)	*p*-values	β (95% CI)	*p*-values	β (95% CI)	*p*-values	*R*^*2*^change
Global Cognition- MMSE	0.276 (-0.117 to 0.669)	0.169	0.498 (-0.696 to 1.692)	0.413	-0.36 (-0.67 to -0.049)	0.023*	0.006
Digit span forward	0.237 (-0.302 to 0.776)	0.388	0.617 (-1.019 to 2.253)	0.459	-0.254 (-0.68 to 0.172)	0.242	0.002
Digit span backward	0.087 (-0.353 to 0.528)	0.697	-0.191 (-1.528 to 1.146)	0.779	-0.165 (-0.513 to 0.183)	0.351	0.001
CTT 1	-8.015 (-12.086 to -3.945)	<0.001***	-29.498 (-41.857 to -17.139)	<0.001***	10.78 (7.563 to 13.997)	<0.001***	0.041
CTT 2	-11.886 (-18.737 to -5.034)	0.001*	-34.343 (-55.144 to -13.542)	0.001*	14.643 (9.228 to 20.058)	<0.001***	0.027
CTT Interference	0.029 (-0.103 to 0.161)	0.667	0.27 (-0.13 to 0.671)	0.185	-0.068 (-0.173 to 0.036)	0.197	0.002

Notes: *MMSE* mini-mental state examination, *CTT* color trail test, *TUG* timed up and go test, *FGS* fast gait speed, *30s-CST* 30-second chair stand test, *β* unstandardized beta-coefficient, *95% CI* 95% confidence interval

* indicates *p*<0.05, ** indicates *p*<0.01, and *** indicates *p*<0.001

All the statistical models here were built on top of the respective adjusted models from Table 3, with the addition of an interaction term between physical performance tests and cognitive status. Reference group was the cognitively normal group. For TUG and FGS, a higher score indicates lower performance. Whereas for 30s-CST, a higher score indicates higher performance. For all cognitive tests, a higher score indicates better cognitive performance, except for only Color Trails Test (CTT) 1 & 2, in which a higher score indicates lower cognitive performance

For 30 s-CST, only the association with CTT1 was moderated by cognitive status, in that higher 30 s-CST was associated with lower CTT1 (β = -0.735, 95% CI = -1.33 to -0.141, *p* = 0.015, R^2^ change = 0.7%) (Table 6 and Fig. 3a, b and c).

**Table 6:** Associations between 30s-CST and executive function-based neurocognitive tests– added interaction term between 30s-CST and cognitive status

Neurocognitive tests/ physical performance tests	30s-CST Interaction Model						
	30s-CST		Cognitive Status		30s-CST x Cognitive Status		
	β (95% CI)	*p*-values	β (95% CI)	*p*-values	β (95% CI)	*p*-values	*R*^*2*^change
Global Cognition- MMSE	0.066 (-0.009 to 0.140)	0.084	-0.272 (-1.074 to 0.530)	0.506	-0.042 (-0.097 to 0.013)	0.136	0.002
Digit span forward	-0.012 (-0.114 to 0.09)	0.812	-0.621 (-1.717 to 0.474)	0.266	0.022 (-0.053 to 0.098)	0.559	0.000
Digit span backward	-0.002 (-0.085 to 0.082)	0.972	-0.893 (-1.79 to 0.003)	0.051	0.007 (-0.055 to 0.068)	0.834	0.000
CTT 1	0.411 (-0.393 to 1.214)	0.316	20.289 (11.662 to 28.916)	<0.001***	-0.735 (-1.33 to -0.141)	0.015*	0.007
CTT 2	0.493 (-0.838 to 1.825)	0.467	30.478 (16.181 to 44.775)	<0.001***	-0.785 (-1.771 to 0.201)	0.118	0.002
CTT Interference	0 (-0.024 to 0.025)	0.972	-0.136 (-0.403 to 0.131)	0.319	0.012 (-0.007 to 0.03)	0.211	0.002

Notes: *MMSE* mini-mental state examination, *CTT* color trail test, *TUG* timed up and go test, *FGS* fast gait speed, *30s-CST* 30-second chair stand test, *β* unstandardized beta-coefficient, *95% CI* 95% confidence interval

* indicates *p*<0.05, ** indicates *p*<0.01, and *** indicates *p*<0.001

All the statistical models here were built on top of the respective adjusted models from Table 3, with the addition of an interaction term between physical performance tests and cognitive status. Reference group was the cognitively normal group. For TUG and FGS, a higher score indicates lower performance. Whereas for 30s-CST, a higher score indicates higher performance. For all cognitive tests, a higher score indicates better cognitive performance, except for only Color Trails Test (CTT) 1 & 2, in which a higher score indicates lower cognitive performance

As shown in Supplementary Table 4a, b and c, for the non-EF-based cognitive tests, after fully controlling for covariates, cognitive status had no significant moderation effects on most associations with TUG, except Semantic Fluency (Animal) Test (β = 0.321, 95% CI = 0.049 to 0.594, *p* = 0.021, R^2^ change = 0.6%. After fully controlling for covariates, cognitive status remained having significant moderation effect (with higher associations in MCI vs CN) on the associations between FGS/30 s-CST and two neurocognitive tests, i.e. RAVLT Recognition Trial (β = 0.384, 95% CI = 0.114 to 0.653, *p* = 0.005, R^2^ change = 0.9%; β = -0.055, 95% CI = -0.103 to -0.007, *p* = 0.024, R^2^ change = 0.6%, respectively), and Block Design test (β = -2.092, 95% CI = 3.679 to 0.505, *p* = 0.010, R^2^ change = 0.6%; β = 0.312, 95% CI = 0.029 to 0.594, *p* = 0.031, R^2^ change = 0.4%, respectively).

Taken together Tables 4 and 5, Figs. 1 and 2, and Supplementary Table 4, several associations between all three physical performance tests and neurocognitive tests were highly contingent on cognitive status. Across most of the significant associations, MCI had the higher strength of association compared to CN, in that lower physical measures were significantly and more strongly associated with lower cognitive tests than CN and vice versa.

## Discussion

This study presents three notable findings. First, of the three physical performance tests examined, only TUG was significantly associated with global cognition. With detailed neurocognitive tests, upon controlling for covariates and multiple testing, all three physical performance tests only strongly associated with EF-based cognitive tests, but not memory-based neurocognitive tests. Second, there were differential associations between the physical performance measures and global and EF-based neurocognitive functions. Specifically, TUG had the highest number of and the strongest significant associations with neurocognitive tests, followed by the FGS, and lastly, 30 s-CST. Notably, TUG was not only associated with global cognition, but it also had significant and robust associations with multiple EF-based neurocognitive tests, encompassing executive functions, information updating and monitoring, and mental shifting. Notably, TUG was also most strongly and positively associated with the more cognitively demanding neurocognitive tests which taps on higher-order cognitive domains, instead of the lower domains. Third, we showed pilot data on the significant moderating effect of cognitive status on the associations between all three simple physical performance tests and multiple EF and non-EF-based neurocognitive tests, in that several associations between three physical performance tests and neurocognitive tests were highly contingent on cognitive status. Across most of the significant associations, MCI had the higher strength of and positive association compared to CN, in that lower physical measures were significantly and more strongly associated with lower cognitive performance than CN. Hence, our findings suggest that despite the absence of significant differences in the three physical performance tests between CN and MCI, given their differential associations with global and detailed neurocognitive tests and moderations by cognitive status, there is a need to carefully consider the choices of simple physical performance tests when using these tests with a heterogenous group of community-dwelling older adults without dementia.

There were differential associations between the three simple physical performance tests with the EF-based neurocognitive measures. Intriguingly, only the TUG was significantly and strongly associated with global cognition, but not the FGS and 30 s-CST. For the detailed neurocognitive tests, there were differential associations between physical performance measures and the EF-based neurocognitive functions. All three physical performance tests were strongly associated with the CTT tests. These findings align with previous studies showing that low physical performances were strongly associated with low EF scores [20, 27, 50, 51], implying diminished executive controls involving motor-controls and speed components. Amongst the three physical performance tests, TUG was significantly and strongly associated with the highest number of EF-based neurocognitive tests. They encompass executive functions, information updating and monitoring, and mental shifting, suggesting that the involvements of multiple EF-based cognitive domains are necessary to execute transfer, turning and walking in this complex task, concurring with previous findings found in *other* populations [52, 53]. Here, we showed the presence of these associations in *both CN and MCI*. Thus, physical tasks involving a lower grade of planning and less complexity in nature, such as the FGS and 30 s-CST, may explain the non-significant and weaker associations with neurocognitive measures [54]. Furthermore, TUG was more strongly associated with the more cognitively demanding cognitive tests, which taps on higher-order cognitive domains, than the less cognitively demanding tasks, such as the digit forward test. As such, complex cognitive processes that involve not only the storage of information in short-term memory but also the manipulation of that information, as is required in the digit span backward test, may be relevant to complex motor coordination and strength required of the TUG.

Extant literature has mostly focused on examining the associations between physical performance and neurocognitive tests in participants diagnosed with neurodegenerative diseases [55]. In this study, we extended the literature by investigating these associations in a sample comprising those at risk of dementia (CN and MCI), comparing head-to-head the presence and the lack of associations between physical performance and EF-based cognitive tests in older adults without dementia. Such an investigation has implications for informing future studies on whether there is a need to separately examine these associations, particularly in heterogeneous community-dwelling samples with varying cognitive status. Amongst the three physical performance tests, we found that there was a significant and strong moderating effect of cognitive status on the associations between all three physical performance tests and neurocognitive tests. Across most of the significant associations, MCI had the higher strength of association compared to CN, in that lower physical measures were significantly and more strongly associated with lower cognitive tests than CN. These findings thus suggest that the strengths *and* the presence of several associations between physical performance tests and neurocognitive tests are contingent on cognitive status, supporting the need for separate examination of their associations in a heterogenous population at risk of developing dementia. For example, the higher strengths of associations between TUG and two EF-based tests in MCI compared to CN suggest that the associations were more tightly coupled in MCI, with a potential higher engagement of EF required to execute the TUG test as one progresses to MCI [5, 20]. As hypothesized, there could be compensatory mechanisms to mitigate the insults caused by the aging processes, vascular damages, and/or the neurodegenerative processes as one progresses from CN to MCI [10]. A higher engagement of EF required to execute physical performance could be explained by the age-associated decline in motor and sensory systems, which lead to a decrease in automaticity, accompanied by the compensatory mechanism of an increased engagement of EF [19]. In this study, we reasoned that by engaging with this compensatory mechanism and thus increased reliance on EF, older adults with MCI attempt to maintain their abilities to process motor sensory inputs. A shared neurobiological substrate for these strongly associated measures in MCI could explain this association; A neuroimaging study shows that white matter hyperintensity load was associated strongly with not just motor control and posture measures, but also EF dysfunctions [56]. Extending beyond this study, a previous longitudinal study showed that physical performance impairment in patients with dementia causes EF dysfunction, leading to central misprocessing of information [19], highlighting plausible causal relationships between the measures. Taken together, given that MCI is common but frequently underdiagnosed in community-dwelling older adults, our findings highlighted despite no significant differences in the three physical performance tests between CN and MCI, the necessity to consider the type of simple physical performance test employed and the baseline cognitive status when examining the associations between physical and neurocognitive tests.

This study has several limitations. The cross-sectional nature of this study does not allow causal inference, and future studies using longitudinal design [57] are thus warranted to test the directionality of the associations, particularly honing in on the moderating effects of cognitive status. The physical performance measures were also limited to the simple tests, especially when we did not have dual-task motor performance tests, rendering us unable to examine the higher-level physical measures. However, the focus of this study was to examine the usefulness of the simple physical tests. Furthermore, the methodology for measuring dual-task motor performance is not yet standardized, with some studies focusing more on one task than the other, which can make the direct comparisons of dual-task motor performance findings across studies relatively difficult [58]. Although unequal group sample sizes in MCI and CN might may have affected the differential associations between MCI and CN, compared to the extant studies that frequently examined such associations *separately* in either MCI or CN in N < 100 older adults, our study is one of the largest with investigation of these associations in *both* CN (N = 562) and MCI (N = 154) participants. Lastly, we did not have a representative sample, and thus our findings have limited generalizability. However, this study addressed the gap in knowledge on the lack of studies examining these associations in this understudied and underrepresented population. Our sample has higher proportions of younger, male and highly-educated older adults, and how these factors could influence the associations between measures could be topics for future investigations.

To the best of our knowledge, this is to date one of the first studies associating three simple physical performance measures and an extensive battery of neurocognitive tests in community-dwelling older adults without dementia. This study contributed pilot data to further our understanding on the granularities in the cognitive processes associated with different physical performances. Second, all the participants in the study were clinically diagnosed during the study’s monthly consensus meeting (involving two senior consultant psychiatrists and a neuropsychologist), overcoming the issue of using cut-offs scores from screening tests employed in previous studies [59], which invariably has its shortcomings in inaccurately classifying cognitive status. Third, our sample population included CN older adults and those diagnosed with MCI, allowing us to examine the associations between physical performance and detailed neurocognitive tests at a modifiable disease stage, overcoming limitations in the extant literature primarily examining these associations in patients with dementia and/or AD. Fourth, we have also shown differential associations between the three physical performance tests with global and detailed neurocognitive tests, highlighting the usefulness of a simple physical performance test, especially the TUG, in the communal setting, with potential positive impacts on public health screening approaches. Lastly, this is the first study to investigate and show pilot data on both statistically significant and clinically meaningful moderating effects of cognitive status on the associations between the measures, especially in less-studied Asian community-dwelling older adults.

In summary, our results showed that amongst the three tests, TUG, and to a lesser extent FGS, were useful physical performance tests in associating with neurocognitive tests in community-dwelling older adults without dementia. Hence, looking for signs of impairments in TUG, and to a lesser extent FGS, could potentially identify older adults at risk of cognitive impairment in the communal setting. Further validation of the longitudinal causal associations and moderating effects of cognitive status between these measures is required. Lastly, owing to the strong associations and shared neural mechanisms with cognitive functions, by improving physical performances, clinicians from different backgrounds, including gerontologists, physiatrists, occupational and physical therapists, could improve physical and cognitive functions concurrently, improving these prominent modifiable factors for dementia and other geriatric syndromes.

## Supplementary Information

**Additional file 1: Supplementary Figure 1.** Fast gait speed measurement.

**Additional file 2: Supplementary Table 1.** Neurocognitive Test and its associated cognitive domain(s) and task description.

**Additional file 3: Supplementary Table 2.** Unadjusted and fully adjusted differences in physical performance tests between cognitively normal and mild cognitive impairment.** Supplementary Table 3. **Associations between physical performance and non-EF neurocognitive tests. **Supplementary Table 4** a. Associations between TUG and non-EF neurocognitive tests – Interaction term between physical performance tests and cognitive status added. **Supplementary Table 4 b.** Associations between FGS and non-EF neurocognitive tests – Interaction term between physical performance tests and cognitive status added. **Supplementary Table 4 c.** Associations between 30s-CST and non-EF neurocognitive tests – Interaction term between physical performance tests and cognitive status added. **Supplementary Table 5.** Differences in demographic characteristics of participants excluded and included in the analyses.

## Data Availability

The datasets generated during and analysed during the current study are not publicly available due to IRB restriction but are available from the corresponding author on reasonable request.
