# Supplementary material for: Differential associations between simple physical performance tests with global and specific cognitive functions in cognitively normal and mild cognitive impairment: a cross-sectional cohort study of Asian community-dwelling older adults
Source: BMC Geriatr. 2022 Oct 13;22:798. doi: 10.1186/s12877-022-03434-4 (PMC9563467; doi:10.1186/s12877-022-03434-4)
Supplement: Supplementary file 1 — Additional file 1: Supplementary Figure 1. Fast gait speed measurement. [file 12877_2022_3434_MOESM1_ESM.docx]

**Supplementary text**

**Timed Up and Go (TUG)**

The TUG test requires participants to stand up and out of the chair, walk 3 m, turn around, walk back to the chair, and sit down. At the beginning of the test, participants were instructed to sit with their backs against an armchair. At the word “go” by the assessor, participants were to get up from the chair, walk at their regular pace for a distance of three meters (indicated by a marking on the floor), turn, walk back to the chair and sit down again [1]. Additionally, participants were instructed to walk with their comfortable and usual walking speed and avoid fast speed walking, with no physical assistance provided to the participants. An untimed practice trial was administered for participants to be familiarized before the actual timed trial, which was completed once. Assessors measured, in seconds, the time taken by participants with a stopwatch; timing began on the word “go”, and timing ended when the participant were seated again on the chair. Assessors also observed participants’ gait performance and ticked all that applied: 1) slow tentative pace, 2) loss of balance, 3) short strides, 4) little or no arm swing, 5) steadying self on walls, 6) shuffling, 7) en-bloc turning, and 8) not using assistive device properly.

**Fast Gait Speed Test (FGS)**

An eight-meter walkway was first measured and marked, with a one-meter distance indicated from the starting and ending lines for acceleration and deceleration. Participants were instructed to stand behind the starting line. At the word “go” by the assessors, participants walked as fast as they could without running until the assessors said “stop”. Assessors measured, in seconds, the time taken by participants to walk the six-meter distance with a stopwatch; timing began after the leading foot crossed the acceleration path, and timing ended after the leading foot crossed the deceleration path. Participants completed the test twice and an average timing was calculated. Gait speed was measured in meters per second, calculated as the distance divided by the average time taken to walk the distance.

Acceleration path

Deceleration path

Walk timed section

**Supplementary Figure 1.** Fast gait speed measurement (please delete if unnecessary)

1m

1m

6m

**30 seconds Chair Stand Test (30s-CST)**

The test was administered using a chair with back support, which was placed against the wall for stability. Before the test, assessors ensured that: 1) participants were seated in the middle of the chair without arm rest, with their backs straight, 2) arms crossed over their chests and placed on their shoulders, and 3) feet approximately shoulder-width apart and placed on the floor at an angle slightly back from the knees. On the word “go” by the assessors, participants rose to a full standing position and returned to a fully seated position as many times as possible in 30 seconds. Following a demonstration by the assessors, participants practised with one to two repetitions to ensure proper form. The timed trial began afterwards, and participants completed the test once. The timing of the test began with the word “go,” and assessors counted the number of full stands completed in 30 seconds. If participants were more than halfway to a standing position at the end of 30 seconds, it was counted as a full stand. Assessors also noted modifications made to the test, if any, e.g. adjusting the chair height or providing assistance to participants. If the participant required to use his/her arms to stand, the test is ended immediately and scored 0[2,3].

**Mini Mental State Examination (MMSE)**

Commonly-used dementia screening tools, which enables the assessors to estimate the severity of cognitive impairment, as well as to track the course of cognitive changes over time. Each correct answer was awarded one point, with no points given for incorrect answers, or if the participant was unable to answer a question/perform an action.

**Geriatric Depression Scales (GDS)**

GDS consists of 15-item and was used to assess depressive symptoms. The participants rated 15 items with either Yes (1) or No (0) response categories [4]. The score ranges from zero to 15, with a higher score indicating a higher depressive symptom. This scale has good psychometric properties in the Chinese population, with good internal consistency (Cronbach alpha of 0.83) [5].

**Geriatric Anxiety Inventory (GAI)** GAI consists of 20-item and was used to assess anxiety symptoms [6]. The participants rated the items with either agree (1) or disagree (0). The score ranges from zero to 20, with a higher score indicating a higher anxiety symptom. The GAI has good psychometric properties in the Chinese population, with excellent internal consistency (Cronbach alpha of 0.93) [7].

**Reference**

[1] Podsiadlo D, Richardson S (1991) The timed “Up & Go”: a test of basic functional mobility for frail elderly persons. *Journal of the American geriatrics Society* **39**, 142–148.

[2] Rikli RE, Jones CJ (1999) Functional fitness normative scores for community-residing older adults, ages 60-94. *Journal of aging and physical activity* **7**, 162–181.

[3] Jones CJ, Rikli RE, Beam WC (1999) A 30-s chair-stand test as a measure of lower body strength in community-residing older adults. *Research quarterly for exercise and sport* **70**, 113–119.

[4] Yesavage JA, Brink TL, Rose TL, Lum O, Huang V, Adey M, Leirer VO (1982) Development and validation of a geriatric depression screening scale: a preliminary report. *Journal of psychiatric research* **17**, 37–49.

[5] Nyunt MSZ, Fones C, Niti M, Ng T-P (2009) Criterion-based validity and reliability of the Geriatric Depression Screening Scale (GDS-15) in a large validation sample of community-living Asian older adults. *Aging and Mental Health* **13**, 376–382.

[6] Pachana NA, Byrne GJ, Siddle H, Koloski N, Harley E, Arnold E (2007) Development and validation of the Geriatric Anxiety Inventory. *International psychogeriatrics* **19**, 103–114.

[7] Yan Y, Xin T, Wang D, Tang D (2014) Application of the Geriatric Anxiety Inventory-Chinese Version (GAI-CV) to older people in Beijing communities. *International psychogeriatrics* **26**, 517.
