# Supplementary material for: Differential associations between simple physical performance tests with global and specific cognitive functions in cognitively normal and mild cognitive impairment: a cross-sectional cohort study of Asian community-dwelling older adults
Source: BMC Geriatr. 2022 Oct 13;22:798. doi: 10.1186/s12877-022-03434-4 (PMC9563467; doi:10.1186/s12877-022-03434-4)
Supplement: Supplementary file 2 — Additional file 2: Supplementary Table 1. Neurocognitive Test and its associated cognitive domain(s) and task description. [file 12877_2022_3434_MOESM2_ESM.docx]

**Supplementary Table 1. Neurocognitive Test and its associated cognitive domain(s) and task description**

| **Neurocognitive Test** | **Cognitive Domain(s)** | **Task Description** |
| --- | --- | --- |
| RAVLT[1] | Declarative verbal learning and memory (immediate, delayed, and recognition) | Participants were given a list of 15 unrelated words (list A) to learn and immediately recall aloud over five learning trials (Immediate Recall). Subsequently, an interference list of 15 unrelated words (list B) was presented only once for the participants to learn and recall immediately. After which, participants were instructed to recall aloud the words from list A. Approximately 30 minutes later, they were again asked to recall aloud the words from list A (Delayed Recall). Finally, participants were given a list of 50 words, comprising list A, list B, and 20 new distractor words, from which they had to identify the original 15 words (Recognition).  Eight outcome measures were used in RAVLT. RAVLT T1 and RAVLT T5 referred to the total number of words correctly recalled in the first and fifth learning trials from list A during Immediate Recall. RAVLT B referred to the total number of words correctly recalled from the interference list. RAVLT T6 referred to the total number of words correctly recalled from list A during Delayed Recall. Lastly, RAVLT Recognition Trial and RAVLT Recognition Trail – False Positive referred to the total number of words correctly identified and falsely identified from list A during Recognition. |
| Digit Span Forward and Backward Task | Attention and working memory | The Digit Span Forward and Backward Task are subtests from the Wechsler Adult Intelligence Scale III (WAIS-III) [2]. A series of numbers were read aloud by the assessor, of which participants were required to repeat the series of numbers in the same (forward) or reverse (backward) order. The forward trial measures working memory span specifically, while the backward trial involved manipulation of information in the working memory. Two outcome measures were used in the Digit Span Task. Forward and Backward scores were obtained from the total number of forward and backward trials successfully repeated by the participants, respectively. |
| CTT[3] | Divided attention | The CTT consists of two parts. In the first (CTT1), participants connected a series of numbers that were printed within pink and yellow circles, sequentially from 1 to 25. In the second part (CTT2), participants similarly connected the numbers from 1 to 25, but alternated between choosing numbers in either pink or yellow circles. Three outcome measures were obtained from CTT – completion time for CTT1 and CTT2, and interference effect (i.e. CTT interference), which was calculated as the difference in completion times between CTT1 and CTT2, divided by CTT1. |
| Block Design Test | Visuospatial function | The Block Design Test is a subtest from the WAIS-III[2]. Here, participants were instructed to arrange blocks with red and white patterns on different sides to match the required block patterns in each trial. Scoring for the block design test depended on both the accuracy in matching the patterns and speed. Additional points were awarded to participants if they completed the trials within various time limits. |
| Semantic Fluency (Animal) Test[4] | Verbal fluency | Participants were instructed to name as many different animals as they could in one minute. The total score was indicated by the total number of correct and unique animal names. |

Notes: RAVLT=Rey Auditory Verbal Learning Test; CTT=Color Trails Test.

**Reference**

[1] Rey A (1941) L’examen psychologique dans les cas d’encéphalopathie traumatique.(Les problems.). *Arch Psychol*.

[2] Wechsler D (1997) *WAiS-iii*, Psychological Corporation San Antonio, TX.

[3] D’Elia L, Satz P, Uchiyama C, White T (1994) Psychological Assessment Resources.

[4] Lezak MD, Howieson DB, Loring DW, Fischer JS (2004) *Neuropsychological assessment*, Oxford University Press, USA.
