# Supplementary material for: Differential associations between simple physical performance tests with global and specific cognitive functions in cognitively normal and mild cognitive impairment: a cross-sectional cohort study of Asian community-dwelling older adults
Source: BMC Geriatr. 2022 Oct 13;22:798. doi: 10.1186/s12877-022-03434-4 (PMC9563467; doi:10.1186/s12877-022-03434-4)
Supplement: Supplementary file 3 — Additional file 3: Supplementary Table 2. Unadjusted and fully adjusted differences in physical performance tests between cognitively normal and mild cognitive impairment. Supplementary Table 3. Associations between physical performance and non-EF neurocognitive tests. Supplementary Table 4 a. Associations between TUG and non-EF neurocognitive tests – Interaction term between physical performance tests and cognitive status added. Supplementary Table 4 b. Associations between FGS and non-EF neurocognitive tests – Interaction term between physical performance tests and cognitive status added. Supplementary Table 4 c. Associations between 30s-CST and non-EF neurocognitive tests – Interaction term between physical performance tests and cognitive status added. Supplementary Table 5. Differences in demographic characteristics of participants excluded and included in the analyses. [file 12877_2022_3434_MOESM3_ESM.docx]

**Supplementary Table 2. Unadjusted and fully adjusted differences in physical performance tests between cognitively normal and mild cognitive impairment**

| Cognitive status /gait tests | Models | TUG | | | FGS | | | 30s-CST | | |
| --- | --- | --- | --- | --- | --- | --- | --- | --- | --- | --- |
|  |  | β (95% CI) | *P-*values | *R^2^* change | β (95% CI) | *P-*values | *R^2^* change | β (95% CI) | *P-*values | *R^2^* change |
| Cognitive status (cognitively normal/ mild cognitive impairment) | Unadjusted | 0.558 (0.133 to 0.982) | 0.010* | 0.009 | 0.104 (-0.066 to 0.275) | 0.229 | 0.002 | -0.71 (-1.495 to 0.075) | 0.076 | 0.004 |
|  | Fully-adjusted | 0.345 (-0.027 to 0.717) | 0.069 | 0.277 | 0.035 (-0.116 to 0.187) | 0.649 | 0.247 | -0.46 (-1.194 to 0.274) | 0.219 | 0.166 |

Notes: MMSE=mini-mental state examination; TUG=timed up and go test; FGS=fast gait speed; 30s-CST=30-second chair stand test; β=unstandardized beta-coefficient; 95% CI=95% confidence interval; Bold text indicates *p* approximate 0.05, ***** indicates *p*<0.05. Reference group was the cognitively healthy group.

Unadjusted: bivariate association between physical performance and neurocognitive tests

Fully-adjusted: added age, sex, years of formal education, depressive and anxiety symptoms, body-mass index, consumption of prescription medicine, total number of morbidities, physical activity level, smoking status, and alcohol consumption.

**Supplementary Table 3. Associations between physical performance and non-EF neurocognitive tests**

| Neurocognitive tests/gait tests | Models | TUG | | | FGS | | | 30s-CST | | |
| --- | --- | --- | --- | --- | --- | --- | --- | --- | --- | --- |
|  |  | β (95% CI) | *P-*values | *R^2^* change | β (95% CI) | *P-*values | *R^2^* change | β (95% CI) | *P-*values | *R^2^* change |
| RAVLT T1 | Unadjusted | -0.157 (-0.219 to -0.095) | <0.001*** | 0.033 | -0.273 (-0.43 to -0.115) | 0.001** | 0.016 | 0.062 (0.028 to 0.096) | <0.001*** | 0.017 |
|  | Fully-adjusted | -0.03 (-0.097 to 0.038) | 0.389 | 0.161 | -0.078 (-0.244 to 0.088) | 0.356 | 0.178 | 0.025 (-0.009 to 0.06) | 0.145 | 0.178 |
| RAVLT T5 | Unadjusted | -0.119 (-0.185 to -0.053) | <0.001*** | 0.017 | -0.169 (-0.335 to -0.004) | 0.045 | 0.006 | 0.044 (0.008 to 0.079) | 0.017* | 0.008 |
|  | Fully-adjusted | 0.013 (-0.057 to 0.083) | 0.715 | 0.193 | -0.024 (-0.195 to 0.148) | 0.788 | 0.204 | 0.019 (-0.016 to 0.055) | 0.287 | 0.203 |
| RAVLT B | Unadjusted | -0.079 (-0.138 to -0.02) | 0.008** | 0.010 | -0.231 (-0.377 to -0.084) | 0.002** | 0.013 | 0.027 (-0.005 to 0.059) | 0.101 | 0.004 |
|  | Fully-adjusted | 0.018 (-0.049 to 0.084) | 0.604 | 0.087 | -0.105 (-0.268 to 0.057) | 0.204 | 0.086 | -0.001 (-0.034 to 0.033) | 0.963 | 0.093 |
| RAVLT T6 | Unadjusted | -0.189 (-0.276 to -0.102) | <0.001*** | 0.025 | -0.299 (-0.517 to -0.08) | 0.008** | 0.010 | 0.075 (0.027 to 0.122) | 0.002** | 0.013 |
|  | Fully-adjusted | 0.006 (-0.085 to 0.097) | 0.895 | 0.212 | -0.023 (-0.247 to 0.2) | 0.838 | 0.227 | 0.027 (-0.019 to 0.073) | 0.254 | 0.000 |
| RAVLT Sum T1-T5 | Unadjusted | -0.777 (-1.056 to -0.499) | <0.001*** | 0.040 | -1.209 (-1.914 to -0.504) | 0.001** | 0.016 | 0.298 (0.146 to 0.451) | <0.001*** | 0.020 |
|  | Fully-adjusted | -0.069 (-0.349 to 0.212) | 0.631 | 0.266 | -0.23 (-0.919 to 0.458) | 0.511 | 0.290 | 0.12 (-0.022 to 0.262) | 0.097 | 0.288 |
| RAVLT Delayed Recall | Unadjusted | -0.2 (-0.292 to -0.109) | <0.001*** | 0.025 | -0.262 (-0.494 to -0.031) | 0.026* | 0.007 | 0.067 (0.017 to 0.117) | 0.009** | 0.009 |
|  | Fully-adjusted | -0.005 (-0.1 to 0.089) | 0.915 | 0.238 | -0.02 (-0.252 to 0.212) | 0.865 | 0.256 | 0.019 (-0.029 to 0.067) | 0.431 | 0.255 |
| RAVLT Recognition Trial | Unadjusted | -0.085 (-0.135 to -0.035) | 0.001** | 0.016 | -0.085 (-0.209 to 0.04) | 0.183 | 0.002 | 0.032 (0.005 to 0.059) | 0.019* | 0.008 |
|  | Fully-adjusted | -0.017 (-0.07 to 0.037) | 0.538 | 0.172 | 0.017 (-0.115 to 0.148) | 0.804 | 0.186 | 0.015 (-0.012 to 0.042) | 0.265 | 0.181 |
| RAVLT Recognition Trial – False Positive | Unadjusted | 0.186 (0.067 to 0.305) | 0.002** | 0.013 | 0.468 (0.17 to 0.766) | 0.002** | 0.013 | -0.084 (-0.148 to -0.019) | 0.011* | 0.009 |
|  | Fully-adjusted | -0.052 (-0.186 to 0.081) | 0.439 | 0.111 | 0.109 (-0.217 to 0.436) | 0.512 | 0.111 | -0.01 (-0.077 to 0.058) | 0.781 | 0.114 |
| Block Design test | Unadjusted | -1.072 (-1.378 to -0.766) | <0.001*** | 0.062 | -2.875 (-3.636 to -2.114) | <0.001*** | 0.072 | 0.397 (0.229 to 0.566) | <0.001*** | 0.029 |
|  | Fully-adjusted | -0.247 (-0.562 to 0.067) | 0.122 | 0.234 | -0.543 (-1.314 to 0.229) | 0.168 | 0.224 | -0.012 (-0.171 to 0.147) | 0.883 | 0.265 |
| Semantic Fluency (Animal) Test | Unadjusted | -0.277 (-0.412 to -0.142) | <0.001*** | 0.022 | -0.745 (-1.083 to -0.407) | <0.001*** | 0.026 | 0.132 (0.058 to 0.205) | <0.001*** | 0.017 |
|  | Fully-adjusted | 0.043 (-0.099 to 0.186) | 0.553 | 0.208 | -0.038 (-0.387 to 0.312) | 0.833 | 0.203 | 0.012 (-0.06 to 0.084) | 0.747 | 0.213 |

Notes: RAVLT=Rey Auditory Verbal Learning Test; TUG=timed up and go test; FGS=fast gait speed; 30s-CST=30-second chair stand test; β=unstandardized beta-coefficient; 95% CI=95% confidence interval; Bold text indicates *p* approximate 0.05, ***** indicates *p*<0.05, ** indicates *p*<0.01, and *** indicates *p*<0.001.

Unadjusted: bivariate association between physical performance and neurocognitive tests

Fully-adjusted: added age, sex, years of formal education, depressive and anxiety symptoms, body-mass index, consumption of prescription medicine, total number of morbidities, physical activity level, smoking status, alcohol consumption, and cognitive status.

For TUG and FGS, a higher score indicates lower performance. Whereas for 30s-CST, a higher score indicates higher performance. For all cognitive tests, a higher score indicates better cognitive performance, except for only Color Trails Test (CTT) 1 & 2, in which a higher score indicates lower cognitive performance.

**Supplementary Table 4a. Associations between TUG and non-EF neurocognitive tests – Interaction term between physical performance tests and cognitive status added**

| Neurocognitive tests/ physical performance tests | TUG Interaction Model | | | | | | |
| --- | --- | --- | --- | --- | --- | --- | --- |
|  | TUG | | Cognitive Status | | TUG x Cognitive Status | |  |
|  | β (95% CI) | *p*-values | β (95% CI) | *p*-values | β (95% CI) | *p*-values | *R^2^* change |
| RAVLT T1 | -0.142 (-0.313 to 0.029) | 0.104 | -1.789 (-3.1 to -0.479) | 0.008* | 0.092 (-0.037 to 0.222) | 0.161 | 0.002 |
| RAVLT T5 | -0.144 (-0.321 to 0.032) | 0.109 | -2.641 (-3.995 to -1.286) | <0.001*** | 0.13 (-0.004 to 0.263) | 0.057 | 0.004 |
| RAVLT B | 0.029 (-0.14 to 0.197) | 0.738 | -0.251 (-1.542 to 1.04) | 0.703 | -0.009 (-0.137 to 0.118) | 0.888 | 0.000 |
| RAVLT T6 | -0.105 (-0.336 to 0.126) | 0.372 | -3.072 (-4.841 to -1.303) | 0.001** | 0.091 (-0.083 to 0.266) | 0.304 | 0.001 |
| RAVLT Sum T1-T5 | -0.652 (-1.362 to 0.058) | 0.072 | -11.297 (-16.739 to -5.854) | <0.001*** | 0.48 (-0.056 to 1.017) | 0.079 | 0.003 |
| RAVLT Delayed Recall | -0.16 (-0.399 to 0.079) | 0.19 | -3.814 (-5.647 to -1.982) | <0.001*** | 0.127 (-0.053 to 0.308) | 0.167 | 0.002 |
| RAVLT Recognition Trial | -0.143 (-0.278 to -0.008) | 0.038* | -2.519 (-3.554 to -1.484) | 0.001*** | 0.104 (0.002 to 0.206) | 0.046 | 0.005 |
| RAVLT Recognition Trial – False Positive | -0.193 (-0.53 to 0.144) | 0.262 | 0.892 (-1.692 to 3.477) | 0.498 | 0.116 (-0.139 to 0.371) | 0.374 | 0.001 |
| Block Design test | 0.061 (-0.735 to 0.857) | 0.881 | -0.623 (-6.726 to 5.481) | 0.841 | -0.254 (-0.856 to 0.348) | 0.408 | 0.001 |
| Semantic Fluency (Animal) Test | -0.347 (-0.707 to 0.012) | 0.058 | -6.263 (-9.021 to -3.505) | <0.001*** | 0.321 (0.049 to 0.594) | 0.021* | 0.006 |

Notes: RAVLT=Rey Auditory Verbal Learning Test; TUG=timed up and go test; FGS=fast gait speed; 30s-CST=30-second chair stand test; β=unstandardized beta-coefficient; 95% CI=95% confidence interval; ***** indicates *p*<0.05, ** indicates *p*<0.01, and *** indicates *p*<0.001. All the statistical models were built on top of the respective adjusted models from Supplementary Table 3, with the addition of the interaction terms between physical tests and cognitive status. Reference group was the cognitively normal group. For TUG and FGS, a higher score indicates lower performance. Whereas for 30s-CST, a higher score indicates higher performance. For all cognitive tests, a higher score indicates better cognitive performance, except for only Color Trails Test (CTT) 1 & 2, in which a higher score indicates lower cognitive performance.

**Supplementary Table 4b. Associations between FGS and non-EF neurocognitive tests – Interaction term between physical performance tests and cognitive status added**

| Neurocognitive tests/ physical performance tests | FGS Interaction Model | | | | | | |
| --- | --- | --- | --- | --- | --- | --- | --- |
|  | FGS | | Cognitive Status | | FGS x Cognitive Status | |  |
|  | β (95% CI) | *p*-values | β (95% CI) | *p*-values | β (95% CI) | *p*-values | *R^2^* change |
| RAVLT T1 | -0.179 (-0.612 to 0.255) | 0.419 | -1.213 (-2.528 to 0.103) | 0.071 | 0.086 (-0.256 to 0.429) | 0.622 | 0.000 |
| RAVLT T5 | -0.146 (-0.595 to 0.302) | 0.522 | -1.756 (-3.117 to -0.394) | 0.012* | 0.105 (-0.249 to 0.459) | 0.561 | 0.000 |
| RAVLT B | 0.003 (-0.422 to 0.429) | 0.987 | 0.015 (-1.278 to 1.308) | 0.982 | -0.093 (-0.43 to 0.243) | 0.587 | 0.001 |
| RAVLT T6 | -0.314 (-0.899 to 0.27) | 0.291 | -3.098 (-4.872 to -1.324) | 0.001** | 0.249 (-0.213 to 0.711) | 0.29 | 0.001 |
| RAVLT Sum T1-T5 | -0.598 (-2.399 to 1.203) | 0.514 | -7.777 (-13.244 to -2.309) | 0.005** | 0.315 (-1.109 to 1.738) | 0.664 | 0.000 |
| RAVLT Delayed Recall | -0.516 (-1.121 to 0.088) | 0.094 | -4.144 (-5.98 to -2.308) | <0.001*** | 0.424 (-0.053 to 0.902) | 0.082 | 0.004 |
| RAVLT Recognition Trial | -0.432 (-0.773 to -0.091) | 0.013* | -2.933 (-3.968 to -1.898) | <0.001*** | 0.384 (0.114 to 0.653) | 0.005** | 0.009 |
| RAVLT Recognition Trial – False Positive | -0.511 (-1.363 to 0.342) | 0.24 | 0.033 (-2.556 to 2.622) | 0.98 | 0.53 (-0.144 to 1.204) | 0.123 | 0.003 |
| Block Design test | 1.903 (-0.105 to 3.911) | 0.063 | 4.591 (-1.505 to 10.687) | 0.14 | -2.092 (-3.679 to -0.505) | 0.01* | 0.006 |
| Semantic Fluency (Animal) Test | -0.134 (-1.049 to 0.781) | 0.774 | -3.406 (-6.183 to -0.629) | 0.016* | 0.082 (-0.64 to 0.805) | 0.823 | 0.001 |

Notes: RAVLT=Rey Auditory Verbal Learning Test; TUG=timed up and go test; FGS=fast gait speed; 30s-CST=30-second chair stand test; β=unstandardized beta-coefficient; 95% CI=95% confidence interval; ***** indicates *p*<0.05, ** indicates *p*<0.01, and *** indicates *p*<0.001. All the statistical models were built on top of the respective adjusted models from Supplementary Table 3, with the addition of the interaction terms between physical tests and cognitive status. Reference group was the cognitively normal group. For TUG and FGS, a higher score indicates lower performance. Whereas for 30s-CST, a higher score indicates higher performance. For all cognitive tests, a higher score indicates better cognitive performance, except for only Color Trails Test (CTT) 1 & 2, in which a higher score indicates lower cognitive performance.

**Supplementary Table 4c. Associations between 30s-CST and non-EF neurocognitive tests – Interaction term between physical performance tests and cognitive status added**

| Neurocognitive tests/ physical performance tests | 30s-CST Interaction Model | | | | | | |
| --- | --- | --- | --- | --- | --- | --- | --- |
|  | 30s-CST | | Cognitive Status | | 30s-CST x Cognitive Status | |  |
|  | β (95% CI) | *p*-values | β (95% CI) | *p*-values | β (95% CI) | *p*-values | *R^2^* change |
| RAVLT T1 | 0.012 (-0.07 to 0.094) | 0.774 | -1.03 (-1.91 to -0.15) | 0.022* | 0.011 (-0.05 to 0.072) | 0.724 | 0.000 |
| RAVLT T5 | 0.052 (-0.033 to 0.137) | 0.231 | -1.002 (-1.913 to -0.092) | 0.031* | -0.027 (-0.089 to 0.036) | 0.407 | 0.001 |
| RAVLT B | -0.034 (-0.114 to 0.047) | 0.413 | -0.693 (-1.559 to 0.173) | 0.117 | 0.027 (-0.033 to 0.086) | 0.38 | 0.001 |
| RAVLT T6 | 0.043 (-0.067 to 0.154) | 0.443 | -1.984 (-3.171 to -0.796) | 0.001** | -0.013 (-0.095 to 0.068) | 0.749 | 0.000 |
| RAVLT Sum T1-T5 | 0.081 (-0.259 to 0.421) | 0.64 | -6.987 (-10.641 to -3.333) | <0.001*** | 0.032 (-0.22 to 0.284) | 0.805 | 0.000 |
| RAVLT Delayed Recall | 0.077 (-0.037 to 0.192) | 0.185 | -1.926 (-3.156 to -0.696) | 0.002** | -0.047 (-0.132 to 0.037) | 0.273 | 0.001 |
| RAVLT Recognition Trial | 0.083 (0.018 to 0.148) | 0.012* | -0.763 (-1.457 to -0.07) | 0.031* | -0.055 (-0.103 to -0.007) | 0.024* | 0.006 |
| RAVLT Recognition Trial – False Positive | 0.08 (-0.082 to 0.242) | 0.332 | 2.977 (1.242 to 4.711) | 0.001** | -0.073 (-0.193 to 0.047) | 0.232 | 0.002 |
| Block Design test | -0.394 (-0.776 to -0.013) | 0.043 | -7.368 (-11.46 to -3.276) | <0.001*** | 0.312 (0.029 to 0.594) | 0.031* | 0.004 |
| Semantic Fluency (Animal) Test | -0.066 (-0.239 to 0.107) | 0.452 | -3.948 (-5.805 to -2.09) | <0.001*** | 0.064 (-0.064 to 0.192) | 0.33 | 0.001 |

Notes: RAVLT=Rey Auditory Verbal Learning Test; TUG=timed up and go test; FGS=fast gait speed; 30s-CST=30-second chair stand test; β=unstandardized beta-coefficient; 95% CI=95% confidence interval; ***** indicates *p*<0.05, ** indicates *p*<0.01, and *** indicates *p*<0.001. All the statistical models were built on top of the respective adjusted models from Supplementary Table 3, with the addition of the interaction terms between physical tests and cognitive status. Reference group was the cognitively normal group. For TUG and FGS, a higher score indicates lower performance. Whereas for 30s-CST, a higher score indicates higher performance. For all cognitive tests, a higher score indicates better cognitive performance, except for only Color Trails Test (CTT) 1 & 2, in which a higher score indicates lower cognitive performance.

| Demographics Characteristics | Excluded;  mean ± SD or  n (%) | Included;  mean ± SD or  n (%) | *P*-values |
| --- | --- | --- | --- |
| Sample Size | 115 | 716 | - |
| Age (in years) | 71.63 ± 6.872 | 68.01 ± 5.969 | <0.001*** |
| Sex | | | |
| Women | 54 (77.1%) | 473 (66.1%) | 0.063 |
| Men | 16 (22.9%) | 243 (33.9%) |  |
| Years of formal education | 10.421 ± 5.265 | 13.047 ± 4.25 | <0.001*** |
| Depressive symptoms | 1.12 ± 1.748 | 1.08 ± 1.886 | 0.851 |
| Anxiety symptoms | 1.24 ± 2.6 | 1.22 ± 2.69 | 0.942 |
| BMI (kg/m^2^) | 20.667 ± 4.565 | 23.754 ± 3.772 | 0.158 |
| Smoking status | | | |
| Yes | 1 (1.4%) | 13 (1.8%) | 1 |
| No | 71 (98.6%) | 702 (98.2%) |  |
| Alcohol consumption | | | |
| Yes | 8 (11.1%) | 142 (19.9%) | 0.083 |
| No | 64 (88.9%) | 573 (80.1%) |  |

**Supplementary Table 5. Differences in demographic characteristics of participants excluded and included in the analyses**
